# SOCIAL TIES AND SUICIDAL IDEATION AMONG OLDER ADULTS REFERRED FOR A BEHAVIORAL HEALTH EVALUATION

**DOI:** 10.1093/geroni/igz038.997

**Published:** 2019-11-08

**Authors:** Shahrzad Mavandadi, Erin Ingram, Johanna Klaus, David Oslin

**Affiliations:** Corporal Michael J Crescenz VA Medical Center, Philadelphia, Pennsylvania, United States

## Abstract

The association between the quality and nature of social relationships and suicidal outcomes across the lifespan is well established. However, social ties are rarely assessed in primary care and other clinical settings where older adults at high risk for suicide-related outcomes are seen. This study examined the unique associations between three indices of social ties (i.e., perceived social support, frequency of negative social exchanges, and degree of social integration) and death/suicidal ideation among 3,261 older veterans (aged 65+) who completed a clinical mental health/substance use (MH/SU) assessment upon referral to a Primary Care-Mental Health Integration (PCMHI) program. Data on sociodemographics, MH/SU conditions (e.g., depression, anxiety, and substance use), perceived health, the three indices of social ties, and death/suicidal ideation were extracted from clinical interviews. Veterans were on average 70.8 years old (+6.5 years) and primarily male. Approximately half were married and 60.3% were non-Hispanic white. Forty percent reported death ideation or suicidal ideation, as measured by the Paykel Suicide Scale. Logistic regression analyses revealed that, adjusting for covariates, while perceived social support was associated with a greater risk of reporting death ideation relative to no ideation, both social support and frequency of negative exchanges were uniquely associated with greater risk of reporting suicidal ideation relative to no ideation. Social integration was unrelated to odds of death or suicidal ideation. Findings underscore the value of integrating assessments of multiple aspects of social ties into routine PCMHI practice, as doing so has the potential to enhance suicide screening and intervention efforts.

